# ^18^F-FDG PET-guided diffusion tractography reveals white matter abnormalities around the epileptic focus in medically refractory epilepsy: implications for epilepsy surgical evaluation

**DOI:** 10.1186/s41824-020-00079-7

**Published:** 2020-06-25

**Authors:** Stefan E. Poirier, Benjamin Y. M. Kwan, Michael T. Jurkiewicz, Lina Samargandy, David A. Steven, Ana Suller-Marti, Victor Lam Shin Cheung, Ali R. Khan, Jonathan Romsa, Frank S. Prato, Jorge G. Burneo, Jonathan D. Thiessen, Udunna C. Anazodo

**Affiliations:** 1 Lawson Imaging, Lawson Health Research Institute, 268 Grosvenor St., London, Ontario N6A 4 V2 Canada; 2 Department of Medical Biophysics, Schulich School of Medicine and Dentistry, Western University, London, Ontario Canada; 3 Department of Diagnostic Radiology, Queen’s University, Kingston, Ontario Canada; 4 Department of Medical Imaging, Schulich School of Medicine and Dentistry, Western University, London, Ontario Canada; 5 Epilepsy Program, Department of Clinical Neurological Sciences, Schulich School of Medicine and Dentistry, Western University, London, Ontario Canada; 6 Department of Epidemiology and Biostatistics, Schulich School of Medicine and Dentistry, Western University, London, Ontario Canada; 7 Li Ka Shing Knowledge Institute, St. Michael’s Hospital, Toronto, Ontario Canada; 8 Imaging Research Laboratories, Robarts Research Institute, London, Ontario Canada

**Keywords:** PET/MRI, Medically refractory epilepsy, Fluorodeoxyglucose, Asymmetry index, Diffusion tractography

## Abstract

**Background:**

Hybrid PET/MRI can non-invasively improve localization and delineation of the epileptic focus (EF) prior to surgical resection in medically refractory epilepsy (MRE), especially when MRI is negative or equivocal. In this study, we developed a PET-guided diffusion tractography (PET/DTI) approach combining ^18^F-fluorodeoxyglucose PET (FDG-PET) and diffusion MRI to investigate white matter (WM) integrity in MRI-negative MRE patients and its potential impact on epilepsy surgical planning.

**Methods:**

FDG-PET and diffusion MRI of 14 MRI-negative or equivocal MRE patients were used to retrospectively pilot the PET/DTI approach. We used asymmetry index (AI) mapping of FDG-PET to detect the EF as brain areas showing the largest decrease in FDG uptake between hemispheres. Seed-based WM fiber tracking was performed on DTI images with a seed location in WM 3 mm from the EF. Fiber tractography was repeated in the contralateral brain region (opposite to EF), which served as a control for this study. WM fibers were quantified by calculating the fiber count, mean fractional anisotropy (FA), mean fiber length, and mean cross-section of each fiber bundle. WM integrity was assessed through fiber visualization and by normalizing ipsilateral fiber measurements to contralateral fiber measurements. The added value of PET/DTI in clinical decision-making was evaluated by a senior neurologist.

**Results:**

In over 60% of the patient cohort, AI mapping findings were concordant with clinical reports on seizure-onset localization and lateralization. Mean FA, fiber count, and mean fiber length were decreased in 14/14 (100%), 13/14 (93%), and 12/14 (86%) patients, respectively. PET/DTI improved diagnostic confidence in 10/14 (71%) patients and indicated that surgical candidacy be reassessed in 3/6 (50%) patients who had not undergone surgery.

**Conclusions:**

We demonstrate here the utility of AI mapping in detecting the EF based on brain regions showing decreased FDG-PET activity and, when coupled with DTI, could be a powerful tool for detecting EF and assessing WM integrity in MRI-negative epilepsy. PET/DTI could be used to further enhance clinical decision-making in epilepsy surgery.

## Introduction

Medically refractory epilepsy (MRE) affects approximately 30% of epilepsy patients and is defined as a chronic neurological disorder where seizures persist despite administration of anti-epileptic drugs (AEDs) (Helmstaedter et al. 2003; Richardson et al. 2004; Jiang et al. 2017). In some MRE patients, surgical resection of the epileptic focus (EF)—the brain region responsible for seizures—can alleviate seizure occurrence and improve overall quality of life (Richardson et al. 2004; Caciagli et al. 2014; Cahill et al. 2019). Positive surgical outcomes are highly dependent on accurate identification of the EF to ensure the epileptic region is safely removed without harming surrounding healthy brain tissue (Bettus et al. 2009). The current gold standard for identifying the EF is intracranial electroencephalography (IC-EEG), where either subdural or depth electrodes are used to directly locate abnormal brain activity (suspected EF) before surgical resection is performed (Knowlton 2006; Blount et al. 2008). However, about 50% of MRE patients continue to have seizures after surgery (Téllez-Zenteno et al. 2005; de Tisi et al. 2011). Surgery can fail to prevent seizures when the EF is not properly delineated or detected prior to resection. Additionally, poor surgical outcomes can occur due to unknown interactions between the EF and surrounding neural networks (Aparicio et al. 2016).

Recent advances in medical imaging have seen the increased clinical use of magnetic resonance imaging (MRI) and positron emission tomography (PET) to non-invasively locate the EF and map out the structure and function of surrounding brain regions. Anatomical MRI can detect structural lesions responsible for seizures in about 60% of MRE patients (Burneo et al. 2015), while other advanced MRI techniques, such as diffusion tensor imaging (DTI), can be used to effectively characterize the EF and its relationships with surrounding brain regions (Aparicio et al. 2016; Jiang et al. 2017). DTI non-invasively characterizes tissue microstructure by providing a three-dimensional model of water diffusion in the brain (Basser and Jones 2002; Jones and Cercignani 2010). In addition, DTI can be used to investigate the structural connectivity of neural networks through mapping out diffusion along white matter (WM) fiber pathways (Le Bihan et al. 1986; Le Bihan 2006; Aparicio et al. 2016; Sivakanthan et al. 2016). WM pathways can be characterized using DTI-derived parameters, which are extracted from the diffusion tensor used to model water diffusion at each voxel in the brain. The most commonly used tensor-derived scalar is fractional anisotropy (FA), which is a measure of WM integrity and describes the tendency of water to preferentially diffuse along the length of the fiber bundle (Le Bihan 2006; Mori and Zhang 2006; Soares et al. 2013). Recent DTI studies have revealed that severe FA reduction in WM may correspond to widespread microstructural abnormalities in MRE (Labate et al. 2015; Jiang et al. 2017). To further assess tissue microstructure breakdown, WM pathways can be visualized by reconstructing WM fibers using diffusion tractography. Diffusion tractography techniques continue to be refined and adapted for neurosurgical planning and these techniques have been shown to accurately track WM fibers in temporal lobe regions essential for surgical success (Sivakanthan et al. 2016).

PET, on the other hand, is the most sensitive non-invasive clinical tool for identifying the EF especially in cases where MRI is negative or equivocal (Burneo et al. 2015). ^18^F-Fluorodeoxyglucose PET (FDG-PET) can be used to detect the EF as brain areas showing decreased glucose uptake (glucose hypometabolism) (Sarikaya 2015; Burneo et al. 2015; Aparicio et al. 2016; Cahill et al. 2019). Glucose hypometabolic regions of interest (ROIs) are often identified by visual assessment of FDG-PET images, however, some abnormalities may be missed during this process. Therefore, semi-quantitative approaches such as asymmetry index (AI) mapping have been proposed to aid visual detection of hypometabolic PET ROIs (Henry et al. 1990; Rausch et al. 1994; Van Bogaert et al. 2000; Didelot et al. 2010; Boscolo Galazzo et al. 2016; Anazodo et al. 2018; Kamm et al. 2018; Shang et al. 2018). AI mapping investigates metabolic abnormalities by measuring the voxel-wise difference in cerebral glucose metabolism between hemispheres and has been shown to be a very sensitive biomarker for epileptogenicity (Didelot et al. 2010; Boscolo Galazzo et al. 2016). Using AI to investigate metabolic asymmetries can be useful because the process may be done on individual patients and does not require comparison to a healthy control database.

Recently, it has been shown that multimodal brain imaging combining PET and MRI information may improve seizure site characterization compared to standalone IC-EEG, PET, or MRI (Burneo et al. 2015). Opportunely, this finding coincides with increased availability of advanced imaging systems that combine PET/MRI into an integrated system. Although researchers are starting to implement simultaneous PET/MRI in the clinical setting, the combined use of PET and DTI for presurgical evaluation of epilepsy is yet to be fully investigated. To our knowledge, only two studies to date have assessed whether cortical glucose hypometabolism seen on FDG-PET is related to WM alterations identified by DTI in the brains of MRE patients (Lippé et al. 2012; Aparicio et al. 2016). However, these studies acquired PET and MRI scans at separate timepoints which can introduce registration errors between modalities, making it difficult to accurately detect the seizure onset zone in the brain and assess relationships between PET and MRI findings. Simultaneous acquisition of PET and MRI data using a hybrid PET/MRI scanner acquires both datasets in the same imaging session with intrinsic spatial and temporal registration, potentially improving the accuracy of detecting the EF and may shed new insight into the pathophysiology of MRE. In this hybrid PET/MRI study, we developed a PET-guided diffusion tractography (PET/DTI) approach combining FDG-PET and diffusion MRI to investigate WM integrity in the brains of MRE patients. AI mapping of FDG-PET was used to guide diffusion tractography of WM tracts in MRE patients to better understand structural connectivity of WM fibers affected by glucose hypometabolic regions (suspected EF). WM fibers were also visually inspected by a neurologist to assess the potential clinical impact of PET/DTI on decision-making in epilepsy surgery.

## Materials and methods

### Patients

The study included 14 MRE patients (6 males and 8 females; mean age = 38 ± 14 years) from the London Health Sciences Centre epilepsy monitoring unit (EMU), diagnosed after failing two or more adequate trials of AEDs. Clinical assessment in the EMU included neuropsychological evaluation, prolonged scalp video-EEG, and 1.5 T MRI to localize the EF. Patient demographics and clinical profile are provided in Table 1. Mean epilepsy onset and duration was 23 ± 13 and 15 ± 15 years, respectively. The cohort consisted of 10 MRI-negative and 4 MRI-equivocal MRE patients, determined based on all available diagnostic information (clinical hypothesis, semiology, and 1.5 T MRI reports). All patients provided written informed consent. The study was approved by the University Research Ethics Board and conducted in accordance with the Declaration of Helsinki ethical standards.

**Table 1:** Patient demographics and clinical profile

Patient no.	Sex	Age (year)	Onset/Duration (year)	Seizure frequency	1.5 T MRI	Clinical hypothesis
1	M	52	4/48	–	Left MTS*	Left temporal-frontal lobe
2	M	29	16/13	4/weekk	Left temporal FCD*	Left frontal lobe
3	F	18	14/4	1/month	Unremarkable	Right frontal lobe
4	M	60	41/19	6/year	Unremarkable	Left temporal-frontal lobe
5	F	28	27/1	Unpredictable	Unremarkable	Right temporal lobe
6	M	29	23/6	2–3/month	Unremarkable	Left temporal-frontal lobe
7	F	32	25/7	–	Unremarkable	Left temporal lobe
8	F	36	15/21	1/week	Bitemporal SH*	Right frontal lobe
9	F	45	35/10	5–7/month	Left MTS*	Left temporal lobe
10	M	23	21/2	1–2/month	Unremarkable	Right temporal lobe
11	F	26	17/9	2–3/month	Unremarkable	Right temporal-frontal lobe
12	F	58	21/37	–	Unremarkable	Left temporal lobe
13	F	38	7/31	–	Unremarkable	Right temporal lobe
14	M	55	54/1	–	Unremarkable	Right temporal lobe

*F* female; *FCD* focal cortical dysplasia; *M* male; *MTS* mesial temporal sclerosis; *SH* subcortical heterotopia

*Equivocal finding

^–^Missing data

### Data acquisition

Data were acquired using a 3 T hybrid PET/MRI scanner (Biograph mMR, Siemens Healthineers, Erlangen, Germany) located at the Lawson Health Research Institute. Patients fasted for at least 6 h prior to the study (fasting blood glucose = 4.3 ± 0.6 mmol/L). PET/MRI was acquired immediately after clinical PET/CT scans (net injected dose of FDG = 190 ± 17 MBq, PET/MRI post-injection time = 72 ± 5 min), and the PET/MRI data were used in this study. Serial MRI scans were performed during a 30-min list-mode PET imaging session. An isotropic (1 mm^3^) high resolution T1-weighted MRI and T2-weighted FLAIR MRI were acquired covering the whole brain using a three-dimensional magnetization-prepared rapid gradient-echo sequence (MPRAGE) and fast-spin echo sequence (SPACE) respectively to assess evidence of structural abnormalities (Brant-Zawadzki et al. 1992). Diffusion-weighted imaging (DWI) was acquired using a single-shot echo-planar imaging (EPI) sequence with the following parameters: 2 mm isotropic resolution, 64 contiguous slices, *b* values = 0, 1000 s/mm^2^ and 64 diffusion encoding directions. Two spin-echo images were acquired in opposite phase-encoding directions with *b* values = 0 s/mm^2^ and 6 directions to correct for inherent susceptibility-induced distortions in DWI. The PET data were reconstructed to one image volume (ordered subset expectation maximization algorithm; 3 iterations, 21 subsets, 2 mm full-width at half-maximum (FWHM) Gaussian filter, 2.5 zoom factor, 344 × 344 × 127 matrix and 2.09 × 2.09 × 2.03 mm^3^ voxels). Attenuation correction was performed using an ultrashort echo time MRI sequence and an offline MRI-based attenuation correction approach (RESOLUTE) (Ladefoged et al. 2015).

### DWI preprocessing

Before image preprocessing, all DWI volumes were visually inspected for artifacts to ensure only good quality data were used. DWI data were preprocessed using an in-house image analysis pipeline that incorporated steps from a variety of different image processing software packages (Figure S1). Each patient’s DWI images were first denoised using an optimized non-local means filter (Wiest-Daesslé et al. 2008; Coupé et al. 2008, 2010) in MATLAB (MathWorks®, Natick, MA) followed by subject motion, eddy current, and bias field corrections using FMRIB’s Software Library (FSL) (Woolrich et al. 2009), MRtrix3 (Tournier et al. 2019), and ANTS (Avants et al. 2011), respectively. Tensors were fit to the data using non-linear least-squares estimation in ExploreDTI (Leemans et al. 2009) to generate an FA map. For WM fiber reconstruction, all diffusion tractography steps were performed using MRtrix3. A single fiber WM response function was estimated from the preprocessed DWI data using a spherical harmonics order of 8. The DWI data were upsampled to 1 × 1 × 1 mm^3^ isotropic voxels, and the fiber orientation distribution function was calculated by constrained spherical deconvolution with a spherical harmonics order of 8 and a whole-brain mask to constrain calculations to voxels within the brain. The maximas of the fiber orientation distribution function were then extracted and used to visualize the WM fibers.

### PET data analysis

PET preprocessing steps were completed using FSL, ANTS, and SPM12 (Wellcome Department of Cognitive Neurology, Institute of Neurology, London). For AI mapping, we used the MNI T1 1 mm isotropic image provided by FSL as a template for spatial alignment of patient FDG-PET images. To account for geometric distortions in patient anatomy between hemispheres, this template was made symmetric by flipping it about the sagittal plane and then calculating the mean image of the flipped and unflipped images. Each patient’s FDG-PET data were spatially normalized to the symmetric template using a three-step registration method in ANTS that consisted of linear and non-linear warping transformations that aligned brain structures in the PET image as closely as possible to the template.

A voxel-wise standardized uptake value (SUV) map was calculated using:

$$ \mathrm{SUV}=\frac{C_{\mathrm{PET}}(t)\times \mathrm{BW}}{\mathrm{Dose}} $$

where *C*_PET_(*t*) is the activity concentration in each voxel of the spatially normalized PET image, BW is the patient’s body weight, and Dose is the net injected dose of FDG. The SUV map was smoothed using a FWHM of 2 mm to account for differences in patient anatomy. Each patient’s T1-weighted image was spatially normalized to the symmetric MNI template and then segmented into gray matter (GM), WM, and cerebrospinal fluid tissue probability maps. Because the EF is typically in GM focal regions, we only considered SUV values in voxels with at least 30% GM (based on segmentation of the aligned T1-weighted MRI). The GM SUV maps were then scaled by the individual mean GM SUV in the cerebellum to account for global metabolism effects in the brain (Anazodo et al. 2018). The relative GM SUV (SUVr) map was spatially flipped about the sagittal plane and a voxel-wise AI map was calculated using:

$$ \mathrm{AI}=\frac{\mathrm{I}\hbox{-} \mathrm{fI}}{2\left(\mathrm{I}+\mathrm{fI}\right)}\times 100 $$

where *I* and fI are the unflipped and flipped SUVr images, respectively. To determine significant hypometabolic areas on PET, a Z-score AI (*Z*_AI_) map was calculated using:

$$ {Z}_{\mathrm{AI}}=\frac{X-\mu }{\sigma } $$

where *X* is the voxel intensity in the AI map, *μ* is the mean AI of all GM voxels in the brain, and *σ* is the standard deviation AI of all GM voxels. Because we did not know the exact distribution of AI values in our sample of patients, we scaled *Z*_AI_ by the degrees of freedom (df) in our sample (Crawford and Garthwaite 2012). For our sample of 14 MRE patients, df was 13 therefore we considered *Z*_AI_ < −1.77 to represent significant hypometabolism compared to the contralateral brain region. In each *Z*_AI_ map, the largest focal GM area containing voxels with *Z*_AI_ < −1.77 was extracted as the hypometabolic PET ROI (suspected EF). To validate our AI mapping approach, these PET ROIs were compared against clinical findings on seizure onset area, including clinical hypothesis, scalp video-EEG, clinical reader assessment of PET SUV images, stereo-EEG (SEEG), and surgical outcome (Engel classification and ground-truth histopathology).

### PET/MR image reading

All FDG-PET and MR images were visually inspected by two neuroradiologists (B.Y.M.K. and M.T.J.). FDG-PET was also inspected by a third reader, a nuclear medicine physician (L.S.). FDG-PET was co-registered and overlaid onto MRI. T1-weighted, T2-weighted, and SUV images were visually assessed using a standard clinical imaging software (MI Neurology, SyngoVia, Siemens Healthcare, Erlangen, Germany). To aid visual assessment of PET, semi-quantitative analysis was also included in the image reading through statistical comparison of SUV values with cerebellar normalization to an age-matched healthy control database provided by the software.

### PET-guided diffusion tractography (PET/DTI)

**Fig. 1:**
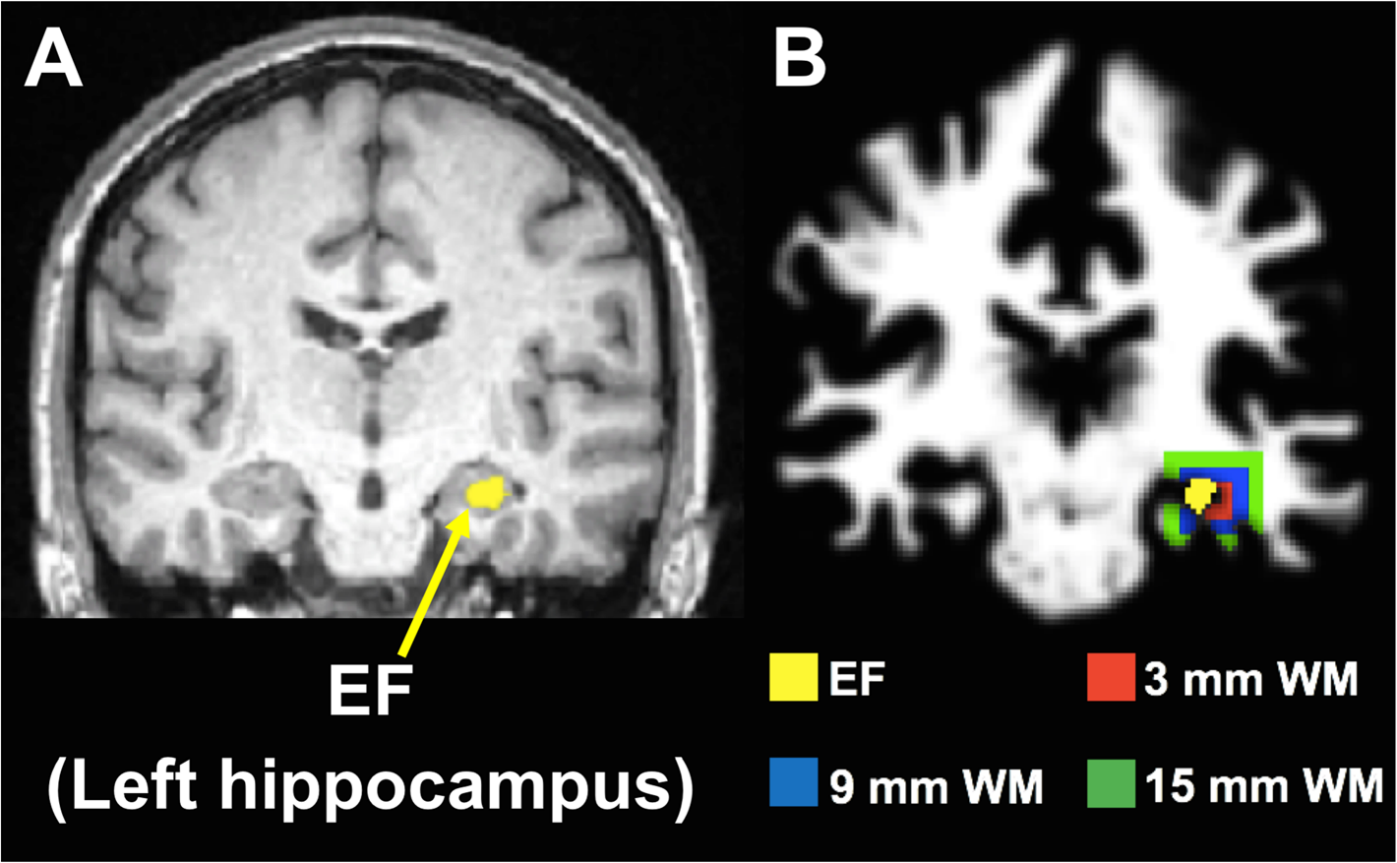
2D representation of the 3D procedure for tracking WM regions around the EF in one MRE patient (patient #9). **a** EF (detected by AI mapping of FDG-PET) overlaid onto structural MRI. **b** EF overlaid onto a WM probability map. Because the EF is located in a cortical area (left hippocampus), WM tracking was performed at three distances away from the EF: 3 mm, 9 mm, and 15 mm. The colored regions around the EF represent WM areas covering the three distances. These WM regions were used as seed ROIs to initiate neural fiber bundle tracking in Fibernavigator

We developed a PET/DTI approach by using seed-based diffusion tractography to investigate structural integrity of WM regions around the hypometabolic PET ROI (suspected EF) identified by AI mapping. The PET ROI, which was initially defined in MNI space, was inverse mapped back to the subject’s diffusion space and used as a seed to initiate fiber tracking. WM fiber tracts were visualized and quantified using Fibernavigator, a novel diffusion tractography tool (Chamberland et al. 2015). In Fibernavigator, a 3 × 3 × 3 mm^3^ volume of interest (VOI) was placed in the GM PET ROI that was directly adjacent to the closest WM area. This VOI was dilated at incremental distances of 3, 9, and 15 mm into surrounding WM (Fig. 1). Each dilated VOI was used as a seed region to generate WM tracts at each distance from the PET ROI. Another 3 × 3 × 3 mm^3^ VOI was manually defined in the contralateral brain region and dilated to generate fibers for the same three distances into surrounding WM. To assess WM tract asymmetry between ipsilateral and contralateral WM fiber tracts, WM fiber quantification was performed by extracting measurements readily available in Fibernavigator, such as fiber count (number of fibers within the bundle), mean fiber length (mm), and mean fiber cross-section (CS) (mm^2^). In addition, the mean FA was calculated as the weighted average of all FA values along the length of the tracts. Normalized (ipsilateral/contralateral) fiber count, mean FA, mean fiber length, and mean CS measurements served as preliminary assessments of WM tract asymmetry and the Wilcoxon signed-rank test was then used to compare fiber measurements across the three WM distances from the PET ROI (*p* < 0.05 was considered significant).

### Clinical assessment of PET/DTI findings

WM fibers around the hypometabolic PET ROI for each patient were visualized by a senior neurologist with over 15 years of practice experience (J.G.B.) in order to assess the potential clinical impact of the PET/DTI approach in guiding epilepsy surgical evaluation. For each patient, the neurologist first viewed the summary of presurgical evaluation findings (clinical hypothesis, scalp video-EEG, 1.5 T MRI, PET report from PET/CT, SEEG) and then using Fibernavigator, interactively viewed the ipsilateral and contralateral WM fibers 3 mm away from the hypometabolic PET ROI identified by AI mapping. A distance of 3 mm away from the PET ROI was chosen for this assessment, as WM fibers generated from this distance pass directly adjacent to the GM PET ROI and are likely to give the best indicator of structural integrity around the epileptic zone. For the clinical assessment of the PET/DTI approach, the neurologist determined whether the differences between ipsilateral and contralateral WM fibers around the hypometabolic PET ROI (suspected EF) were concordant with the clinical hypothesis. In order to assess the potential clinical impact of PET/DTI, the neurologist’s confidence after viewing the WM fibers was assigned to one of the following categories: unchanged or improved. If confidence was improved, the neurologist also reported if reassessment of surgical candidacy would be beneficial in patients who had not undergone surgery.

## Results

### AI mapping of FDG-PET for EF localization and lateralization in MRE

**Fig. 2:**
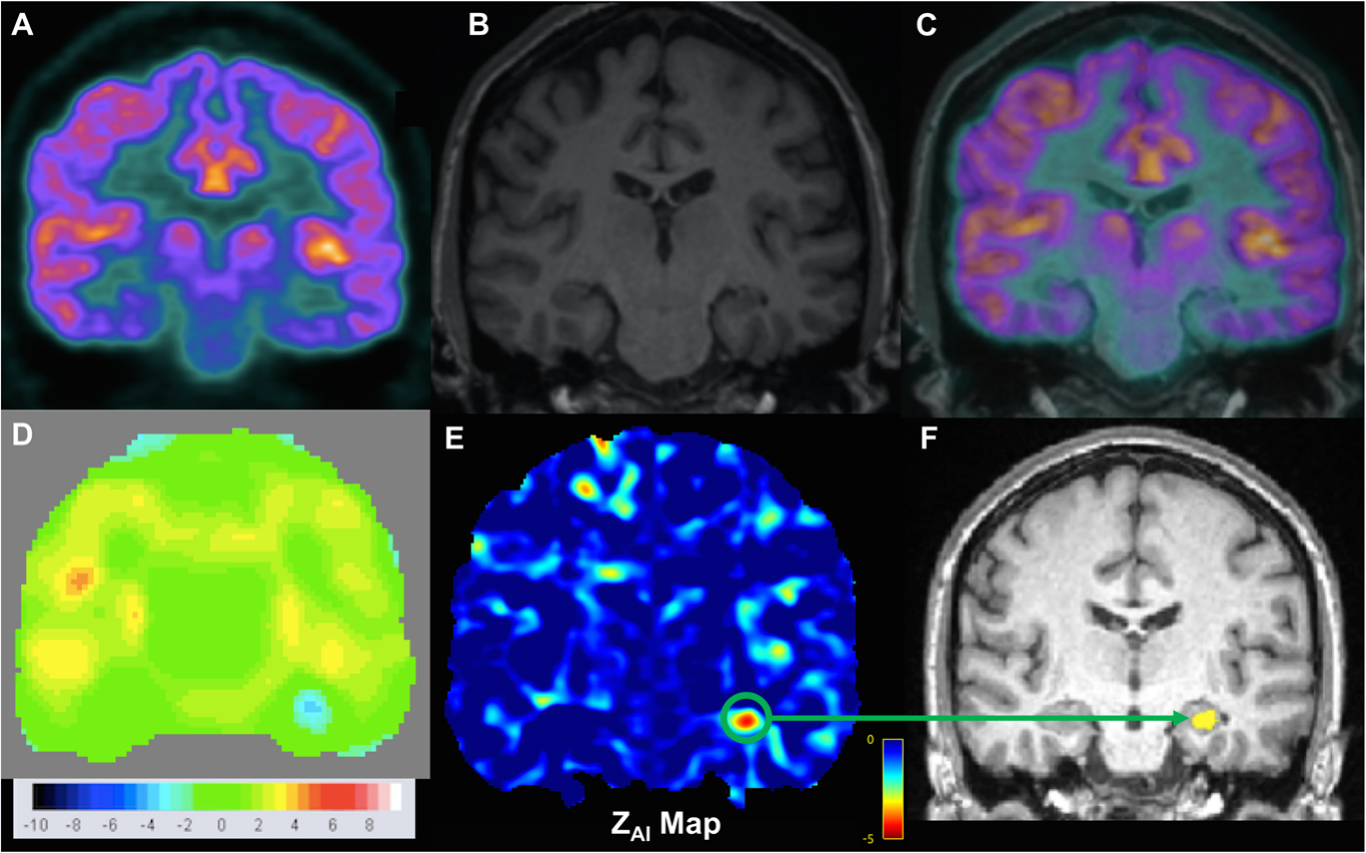
Images from a 45 year old female MRE patient (patient #9) with a clinical hypothesis of left temporal lobe focal epilepsy. **a** PET SUV map. **b** Anatomical MRI. **c** PET fused with MRI. **d** Z-score map from computer-assisted diagnosis of PET data (Siemens Syngo Via). **e** Z-score map generated from AI mapping (Z_AI_ map), which shows a clear glucose hypometabolic region (green circle) in the left temporal lobe, indicative of a potential EF. **f** Hypometabolic PET ROI (yellow) from AI mapping overlaid onto structural MRI

AI mapping was used to detect the EF based on regions showing significant metabolic asymmetry between hemispheres in the brain. A visual example of the AI mapping results for one MRE patient (patient #9) is shown in Fig. 2. In this patient, AI mapping was able to detect a clear hypometabolic region (suspected EF) in the left temporal lobe, which matched the overall clinical hypothesis.

Clinical hypothesis, scalp video-EEG findings from the EMU, FDG-PET hypometabolism reports from the three clinical readers (3 T MRI visual assessment reported in Table S1), AI mapping, SEEG, and surgical findings for our cohort of 14 MRE patients are summarized in Table 2. AI mapping findings were concordant with the clinical hypothesis in localizing and lateralizing the epileptic region in 12/14 (86%) and 9/14 (64%) patients, respectively. AI mapping agreed with scalp video-EEG in 13/14 (93%) patients for both EF localization and lateralization. Concordance between AI mapping and clinical PET readings was 64%/69% (average EF localization/lateralization from the three clinical readers). Five patients underwent SEEG prior to surgical resection, and EF localization/lateralization concordance with AI mapping was observed in four patients. Mean SUV, max SUV, and mean *Z*_AI_ were decreased in hypometabolic PET ROIs identified by AI mapping (see Table S2). Eight patients underwent surgical resection to remove the EF on the suspected epileptogenic side based on all clinical information and diagnoses available. After a one-year follow-up, 5/8 (62.5%) patients achieved Engel class IA (long-term seizure freedom), 2/8 (25%) patients achieved Engel class IIIA (significant improvement, but not completely seizure free), and 1/8 (12.5%) patients had Engel class IV (no improvement). AI mapping was concordant with surgical findings, where histopathology was performed to determine the ground-truth EF classification, in localizing and lateralizing the EF in six and four patients, respectively.

**Table 2:** EEG, PET, and surgical findings

Pt.	Clinical hypothesis	Scalp EEG	PET R1	PET R2	PET R3	AI mapping	PET/DTI	SEEG	Surgery	Engel Class	Histopathology
1	L temp/L front	L temp/L front	Bitemporal (L > R)	L temp	L temp	L temp	L temp	None	None	None	None
2	L front	Bifrontal	L temp/L front/L par	L temp/L front/L par	L front/L par	L front	L front	None	None	None	None
3	R front	Bifrontal	Unremarkable	Unremarkable	Unremarkable	L front	R front	R front	R front lobectomy	IA	GGM WHO I
4	L temp/L front	L temp	Unremarkable	L temp	Bitemporal	L temp	L temp	None	None	None	None
5	R temp	Bitemporal	R temp	R temp	Bitemporal	R temp	R temp	R temp	R temp lobectomy	IIIA	Gliosis
6	L temp/L front	L temp/L front	Bitemporal	L temp/R front	L temp	L front	Unremarkable	L temp	L temp lobectomy	IA	Gliosis
7	L temp	L temp/L front	Bitemporal	Bitemporal	L temp	L front	Unremarkable	None	None	None	None
8	R front	Bitemporal + extratemporal	R temp	R temp/L front	Bitemporal/Bifrontal	L front	Unremarkable	Bifrontal	R front lobectomy	IIIA	FCD Ib
9	L temp	L temp	Bitemporal	Bitemporal	Bitemporal	L temp	L temp	None	L temp lobectomy	IA	HS
10	R temp	R temp	R temp	R temp	R temp	L front	Unremarkable	None	R temp lobectomy	IV	Gliosis
11	R temp/R front	R temp	R temp	R temp/R par/L front	R temp	R temp	R temp	R temp	R temp lobectomy	IA	Gliosis
12	L temp	Bitemporal	L temp	L temp	L temp	L temp	L temp	None	None	None	None
13	R temp	Bitemporal + extratemporal	Bitemporal	Unremarkable	R temp	L temp/L front	R temp	None	None	None	None
14	R temp	Bitemporal	Unremarkable	R temp	R temp	L temp	R temp	None	R temp lobectomy	IA	Unremarkable^a^

FDG-PET hypometabolism reports from three clinical readers are summarized under the headings PET R1, PET R2, and PET R3

Abbreviations: *FCD* focal cortical dysplasia; *front* frontal lobe; *GGM* ganglioglioma; *L* left; *par* parietal lobe; *Pt* patient; *R* right; *temp* temporal lobe; *WHO* World Health Organization grade

^a^No specific structural changes that could explain the etiopathogenesis of patient’s epilepsy

### PET/DTI—tracking WM around glucose hypometabolic regions (suspected EF)

**Fig. 3:**
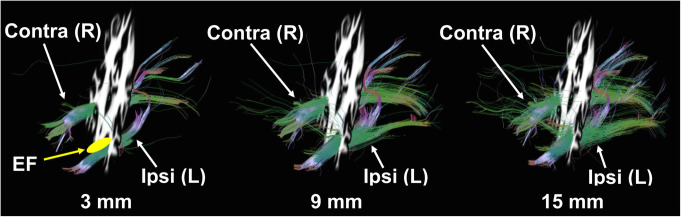
PET-guided diffusion tractography in one MRE patient (patient #9) with a clinical hypothesis of left temporal lobe focal epilepsy. Ipsilateral (left) and contralateral (right) WM fibers (colored lines) are shown for the three WM distances (3, 9, and 15 mm) away from the EF (yellow) identified by AI mapping of FDG-PET. Fewer WM fibers are observed on the ipsilateral side. Differences in WM fibers between ipsilateral and contralateral sides appear more prominent at closer distances (3 mm) to the EF. Abbreviations: L, left; R, right

An example of the WM fiber visualization at each distance away from the hypometabolic PET ROI (suspected EF) for one MRE patient (patient #9) is shown in Fig. 3. In this patient, visual assessment revealed noticeable differences between ipsilateral (left) and contralateral (right) fiber bundles in WM 3 mm away from the EF. No notable differences between ipsilateral and contralateral WM fibers were observed in WM 15 mm away from the EF.

**Fig. 4:**
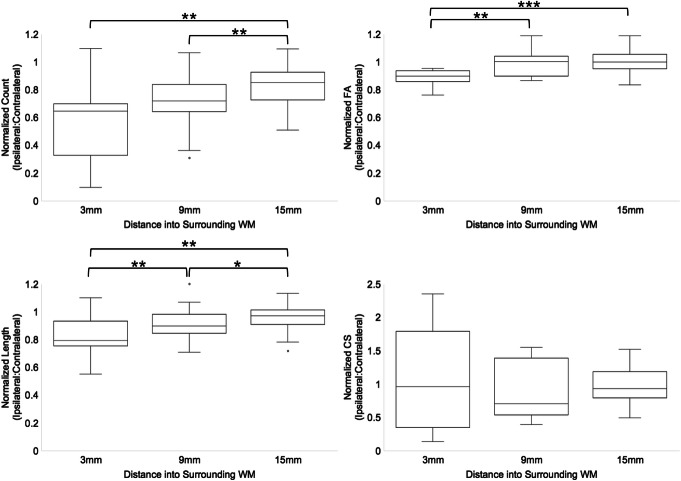
Quantification of WM fibers around the hypometabolic PET ROI (suspected EF) in 14 MRE patients. Ipsilateral fiber measurements were normalized to contralateral fiber measurements as a preliminary measure of WM tract asymmetry. Normalized values are plotted for the three distances away from the PET ROI. Wilcoxon signed-rank test was used to compare normalized fiber measurements across the three distances into surrounding WM (*p* < 0.05 was considered significant). Fiber count, mean fiber length, and mean FA are decreased at closer distances to the PET ROI (3 mm) compared to 15 mm (*p* < 0.05). Abbreviations: *p ≤ 0.05, **p ≤ 0.01, ***p ≤ 0.001

When comparing fiber values across the three distances (3, 9, and 15 mm) into surrounding WM, normalized fiber count, mean FA, and mean fiber length were the lowest at a distance of 3 mm (Fig. 4). At 3 mm, normalized mean FA, fiber count, and mean fiber length were decreased in 14/14 (100%), 13/14 (93%), and 12/14 (86%) patients, respectively. Normalized mean CS was decreased in 7/14 (50%) patients at this same distance. Analysis using the Wilcoxon signed-rank test revealed that mean FA was significantly decreased at 3 mm compared to 9 mm (*p* = 0.0031) and 15 mm (*p* = 0.0004). Fiber count was the lowest at 3 mm and 9 mm, compared to 15 mm (*p* < 0.01). Mean fiber length was significantly reduced across all three distances (*p* < 0.05). The same trend was also observed when DTI scalar measurements were made in the WM seed regions used for tracking around the hypometabolic PET ROI, where mean FA was decreased at distance 3 mm compared to 9 mm and 15 mm (see Table S3).

### Clinical assessment of PET/DTI findings

Table 3 summarizes findings from the neurologist’s clinical assessment of the PET/DTI approach. Eight patients had already undergone surgery. Based on clinical hypotheses, the MRE cohort consisted of seven temporal lobe, four extratemporal lobe, and three frontal lobe epilepsy patients. Upon inspection of PET/DTI, WM fiber abnormalities in the epileptic lobe were observed in 10/14 (71%) patients and these findings were concordant with the clinical hypothesis. In all 10 patients, diagnostic confidence improved after presentation of PET/DTI. Specifically, PET/DTI was contributive in five temporal lobe, three extratemporal lobe, and two frontal lobe epilepsy patients. Most importantly, PET/DTI indicated that surgical resection could be beneficial in 3/6 (50%) patients who had not undergone surgery.

**Table 3:** Clinical assessment of PET-guided diffusion tractography (PET/DTI) findings

Patient	AI mapping	PET/DTI	Confidence after PET/DTI
1	**+**	**+**	**++**
2	**+**	**+**	**++**
3	**+**	**+**	**+**
4	**+**	**+**	**++**
5	**+**	**+**	**+**
6	**+**	**−**	**−**
7	**−**	**−**	**−**
8	**+**	**−**	**−**
9	**+**	**+**	**+**
10	**−**	**−**	**−**
11	**+**	**+**	**+**
12	**+**	**+**	**+**
13	**+**	**+**	**+**
14	**+**	**+**	**+**

Note: EF localization concordance between AI mapping and the clinical hypothesis is reported in the second column

Abbreviations: +, concordant with clinical hypothesis or improved confidence after PET/DTI; ++, PET/DTI improved confidence and indicated that the patient may benefit from an anterior temporal lobectomy; −, discordant with clinical hypothesis or unchanged confidence after PET/DTI

## Discussion and conclusions

To our knowledge, this is the first study to simultaneously combine FDG-PET and diffusion MRI to investigate WM integrity in the brains of MRE patients. We showed that AI mapping of FDG-PET can successfully detect hypometabolic brain regions (suspected EF) that are concordant with conventional epilepsy surgical evaluation techniques (1.5 T MRI, EEG, visual PET assessment). We used AI mapping and diffusion tractography to develop a non-invasive approach that combines PET and MRI information into one integrated tool (PET/DTI). We demonstrated that our PET/DTI approach is feasible and can detect epileptic zones in the brains of MRI-negative epilepsy patients. We localized seizure-onset sites using AI mapping of FDG-PET and tracked WM fibers from these sites to the rest of the brain using diffusion tractography. This was achieved by implementing a robust image analysis process standardized for use in each patient and adapting readily available imaging analysis tools for ROI mask generation and subsequent fiber tracking.

The potential clinical impact of PET/DTI in epilepsy surgical evaluation was also demonstrated in this study. Specifically, we showed that investigation of WM abnormalities adjacent to seizure-onset zones in the brain can improve diagnostic confidence in MRE. Furthermore, we found PET/DTI can even indicate that surgical resection may be beneficial in some MRE patients who have not undergone surgery. Of course, surgical candidacy of these patients would first need to be reassessed through future interdisciplinary meetings before concrete decisions to proceed with the resection can be made. Nevertheless, our findings suggest that PET/DTI can potentially impact clinical decision-making in epilepsy surgery and is a promising tool for advancing epilepsy treatment and management.

Numerous standalone PET and diffusion MRI studies have reported functional and structural alterations in MRE (Henry and Pennell 1998; Knowlton 2006; Focke et al. 2008; Lin et al. 2008; Thivard et al. 2011; James et al. 2015; Labate et al. 2015; Burneo et al. 2015; Sivakanthan et al. 2016; Jiang et al. 2017; Güvenç et al. 2018; Cahill et al. 2019), however, very few studies have assessed relationships between FDG-PET and diffusion MRI findings in epilepsy. Similar to our study, one previous report also found microstructural alterations (decreased FA and increased apparent diffusion coefficient) in WM adjacent to the epileptic zone identified by FDG-PET hypometabolism (Lippé et al. 2012), while another study revealed that metabolic and structural alterations seen using FDG-PET and DTI involve similar brain regions in mesial temporal lobe epilepsy (Aparicio et al. 2016). In contrast to (Lippé et al. 2012) and (Aparicio et al. 2016) who acquired PET and DTI separately, we used a hybrid PET/MRI scanner to simultaneously acquire PET and MRI in our study. While this may appear as a trivial difference, this has profound implications. Patients typically undergo PET and MRI scans on different days, up to a few months apart. In our cohort, the initial 1.5 T MRI evaluation was on average eight months prior to the clinically indicated PET/CT. Acquiring PET and diffusion MRI scans separately can create spatial and temporal registration problems, making it difficult to accurately identify the seizure-onset zone and map its effects on brain structure and function undergoing disease-related changes (Wang et al. 2018; Shang et al. 2018). Misalignment errors are usually due to the subject’s head position being different in image space between scans which are significantly minimized by hybrid PET/MRI. Co-registration of PET with MRI through multimodal imaging therefore may allow for improved diagnostic accuracy and more precise EF detection than standalone PET or MRI, especially in MRI-negative epilepsy (Boscolo Galazzo et al. 2016; Shang et al. 2018).

The majority of the patients with temporal lobe epilepsy in our cohort had apparent PET/DTI WM abnormalities. This result is consistent with past studies that have illustrated the utility of diffusion tractography in revealing microstructural breakdown of WM pathways implicated in drug-resistant temporal lobe epilepsy (Ahmadi et al. 2009; Sivakanthan et al. 2016), as well as other studies reporting FDG-PET to have higher sensitivity for detecting the EF in temporal lobe epilepsy patients (70-90%) who had good surgical outcomes compared to those with other types of epilepsy, especially extratemporal lobe epilepsy (30-60%) (Sarikaya 2015; Burneo et al. 2015; Aparicio et al. 2016). The surgical success rates in extratemporal lobe epilepsy are much lower than temporal lobe epilepsy (30-40% vs. 60-70%) with likelihood of achieving long-term seizure freedom further decreasing in the MRI-negative cases (Téllez-Zenteno et al. 2005; de Tisi et al. 2011), suggesting the possible involvement of intricate neural networks extending beyond the EF in extratemporal lobe epilepsy that may be responsible for surgical failure. Interestingly, PET/DTI identified WM abnormalities around the EF in 3/4 patients with extratemporal lobe epilepsy in our MRE cohort (patients #1, #4, and #11 in Table 3) with improved diagnostic confidence observed in all three patients. While this is a very small number of patients, we argue this might provide some preliminary evidence that PET/DTI may potentially shed new insight into neural networks altered in extratemporal lobe epilepsy and is thus a promising tool for improving surgical outcomes, even in patients where the EF and its interactions with surrounding brain tissue extend beyond the temporal lobe.

In our study, PET/DTI was unremarkable in four patients (see patients #6, #7, #8, and #10 in Table 3). Specifically, in patients #6, #7, and #8, all clinical findings lacked concordance, with only patient #6 becoming seizure-free after surgery (see Table 2). In patient #10, AI mapping was not concordant with visual PET assessment from the three clinical readers and the patient showed no improvement after surgery (Engel class IV). These findings suggest that the four PET/DTI-negative patients in our study may have had a seizure focus with underlying physiological abnormalities that were too subtle to confidently detect using neuroimaging. Further research needs to be conducted on why functional and structural properties measured using PET and MRI are impaired in some epilepsy patients while in others, they appear intact.

It is well established that FDG-PET is the most sensitive functional imaging tool for indirectly identifying epileptic regions based on glucose hypometabolism (Knowlton 2006; Burneo et al. 2015; Aparicio et al. 2016). However, glucose hypometabolic regions identified by PET could extend beyond the true EF especially in extratemporal lobe epilepsy, and may reflect pathophysiology of seizure propagation from the epileptic zone to surrounding neural networks (Sarikaya 2015; Aparicio et al. 2016). Recent studies have found that using semi-quantitative approaches, such as AI mapping that extend beyond visual reads can not only detect hypometabolic regions in high agreement with other clinical and electrophysiological findings but can also increase a reader’s confidence in their visual assessment of PET (Didelot et al. 2010; Boscolo Galazzo et al. 2016; Shang et al. 2018). Here, we demonstrated—albeit retrospectively—the utility of AI mapping in epilepsy surgical evaluation, where AI mapping was able to successfully localize and lateralize the epileptogenic focus in most MRE patients. While it is possible that some of the metabolic asymmetries observed could simply reflect normal physiological asymmetries in the brain, especially in patients with multi-focal hypometabolism, we used a standard AI mapping thresholding approach to isolate significant hypometabolic brain regions that has been validated by past studies (Boscolo Galazzo et al. 2016; Shang et al. 2018), which gives us confidence that the metabolic asymmetries detected in our study are more likely associated with epileptic regions rather than normal healthy brain tissue. AI mapping is thus a promising tool for guiding assessment of surgical candidacy in epilepsy, especially in MRI-negative cases. Furthermore, similar to our findings, past studies have reported FDG-PET hypometabolism in contralateral brain regions in some epilepsy patients (Aparicio et al. 2016; Cahill et al. 2019), presumably due to spread of epileptic activity across hemispheres. Despite these challenges with FDG-PET specificity, we were still able to show that FDG-PET can aid detection of the epileptogenic zone and assessment of surgical candidacy in epilepsy, especially when combined with DTI. Perhaps the use of novel PET tracers targeted to pathogenesis of epilepsy such as imaging reduced synaptic density using PET-ligands targeting the synaptic vesicle protein 2A (Finnema et al. 2016) as well as receptor imaging using PET tracers targeting serotonin and gamma-aminobutyric acid (Sarikaya 2015; Galovic and Koepp 2016), could increase the specificity of PET in detecting the true EF.

In this study, we used diffusion tractography to assess structural integrity around MRI-negative epileptic zones identified by FDG-PET. Although there is no current gold standard for validation of WM fibers generated using diffusion tractography techniques, there are a number of phantom models adapted to simulate WM pathways in healthy human brains and provide some evaluation of tractography approaches. We empirically evaluated our diffusion MRI preprocessing and tractography approach to a computer-simulated WM phantom (Neher et al. 2014). However, this and other phantom models do not take into account any GM or WM pathologies present in epilepsy patients (Neher et al. 2014; Maier-Hein et al. 2017). As such, we opted not to compare WM fibers between epilepsy patients and a healthy control group, and instead assessed structural integrity by comparing WM fibers between hemispheres within individual patients. This individual assessment is more likely to be of clinical utility in epilepsy surgical centers where epilepsy patients are typically evaluated on a case-by-case basis. Nevertheless, we were able to show that WM fibers appear to be affected at multiple distances away from the epileptic tissue. Interestingly, these abnormalities were most apparent in WM directly surrounding the epileptic zone. While no other studies to date have assessed WM fiber integrity at different distances from MRI-negative EF sites using WM fiber quantification, some studies have shown that diffusion tractography can reveal widespread microstructural changes in drug-resistant epilepsy that could be responsible for surgical failure (Sivakanthan et al. 2016; Jiang et al. 2017). Our results suggest that WM directly adjacent to the epileptic zone is most prone to structural alterations. More specifically, we found that out of the three WM distances investigated, WM anomalies were most prominent at an average distance of approximately 3 mm away from the epileptic zone. This finding suggests that investigation of WM at this distance from epileptic tissue may better inform clinicians about whether surgery is an option, and if so, how to properly resect the EF without damaging surrounding healthy brain tissue. This is especially important to assess in WM affecting memory, language, and visual pathways in the brain, which are of prime importance in perioperative planning (Lin et al. 2008; James et al. 2015; Sivakanthan et al. 2016; Li et al. 2019).

Because our AI mapping procedure detected hypometabolism (suspected EF) in cortical brain areas, we were left with the task of developing a method to track surrounding WM regions closest to the EF. We sampled WM regions at three incremental distances away from the epileptic zone using a VOI placed manually in the part of the EF directly adjacent to surrounding WM. This manual implementation poses a few issues. First, because we manually defined VOIs in GM regions contralateral to the EF, there is the possibility of spatial error between ipsilateral and contralateral VOIs. Second, focal cortical dysplasias and other GM/WM pathologies may result in different amounts of WM being sampled between ipsilateral and contralateral regions. However, it is conceivable that any differences in WM size between ipsilateral and contralateral regions are presumably small and are likely offset by the noticeable WM fiber abnormalities observed around the EF in the majority of our MRE patient cohort.

The clinical potential of the proposed PET/DTI approach could be impacted by the relatively small size of our heterogeneous MRE patient cohort, making it difficult to draw any conclusions regarding what epilepsy patient groups are most likely to benefit from PET/DTI. However, the purpose of this hybrid PET/MRI study was to demonstrate the feasibility of PET/DTI and provide some preliminary assessment on whether PET/DTI could potentially impact clinical decision-making in epilepsy surgery, particularly in MRI-negative epilepsy where FDG-PET could instead be used to non-invasively locate the EF. Of note, hybrid PET/MRI relies on MR-based attenuation correction (MRAC) for PET reconstruction instead of CT-based AC used in PET/CT, which is the current clinical standard for FDG-PET imaging in epilepsy. While some studies show that traditional MRAC approaches can produce small bias in quantitative PET due to inadequate modeling of bone (Larsson et al. 2013; Andersen et al. 2014), recent reports have revealed that these MRAC biases do not significantly impact the clinical diagnosis of FDG-PET readings in epilepsy (Paldino et al. 2017; Oldan et al. 2018). Nevertheless, alternative MRAC methods have been proposed to reduce potential bias in reconstructed PET (Ladefoged et al., 2017). In our study, we used an improved robust MRAC method (Ladefoged et al. 2015) that adequately models bone tissue information to produce PET/MR images that provide comparable diagnostic information to PET/CT.

Because the clinical assessment of our PET/DTI approach was retrospectively completed by one neurologist, potential interobserver variability could not be determined from this study. A potential future direction of this research is to pilot a prospective study to assess the clinical utility of combined PET/DTI through interdisciplinary meetings that would evaluate MRE patients both with and without including our PET/DTI approach to determine whether this approach will have any impact on the final surgical decision in these patients.

In general, this retrospective study demonstrated the feasibility of combining PET and DTI to investigate WM integrity in the brains of MRE patients to further enhance clinical decision-making in epilepsy surgery. An extension of this study could combine functional MRI (fMRI) with DTI and PET to map out the structure and function of brain networks in the presence of seizure-related brain abnormalities. fMRI is another non-invasive imaging modality that may have promising applications in neurosurgical planning. While DTI investigates structural connections, fMRI measures functional correlates between brain regions based on differences in blood flow and can be used to effectively map neural connections in the brain (Bettus et al. 2009, 2010; Fox and Greicius 2010; Moeller et al. 2011; Pittau et al. 2012). By combining structural and functional connectivity analysis, we would be able to even better characterize seizure sites in MRE surgical candidates. We plan to incorporate PET, DTI, and fMRI modalities into an integrated software platform that would allow clinicians to non-invasively probe healthy brain tissue and areas around the epileptic zone to further improve neurosurgical planning, especially in challenging epilepsy cases where MRI and IC-EEG findings lack concordance. The integration and proper use of these non-invasive imaging modalities will help advance the field of epilepsy treatment and management and may lead to completely non-invasive epilepsy surgical planning (Knowlton 2006; Sivakanthan et al. 2016).

## Supplementary information

**Additional file 1: Figure S1.** Diffusion MR image analysis pipeline. **Table S1.** EEG and MRI findings from clinical reports and visual assessment. **Table S2.** SUV analysis in hypometabolic PET ROIs and contralateral ROIs from AI mapping in 14 MRE patients. **Table S3.** Regional FA analysis in WM surrounding hypometabolic PET ROIs and contralateral ROIs detected by AI mapping of FDG-PET.

## Data Availability

Data are available from the corresponding authors upon reasonable request and with permission of the Lawson Health Research Institute and Western University Health Research Ethics Board.

## Abbreviations

AED: Anti-epileptic drug
AI: Asymmetry index
ANTS: Advanced normalization tools
BW: Body weight
CS: Cross-section
CT: Computed tomography
df: Degrees of freedom
DTI: Diffusion tensor imaging
DWI: Diffusion-weighted imaging
EEG: Electroencephalography
EF: Epileptic focus
EMU: Epilepsy monitoring unit
EPI: Echo-planar imaging
FA: Fractional anisotropy
FDG: ^18^F-fluorodeoxyglucose
FLAIR: Fluid-attenuated inversion recovery
fMRI: Functional MRI
FSL: FMRIB’s Software Library
FWHM: Full-width at half-maximum
GM: Gray matter
IC-EEG: Intracranial EEG
MNI: Montreal Neurological Institute
MPRAGE: Magnetization-prepared rapid gradient-echo
MRAC: MR-based attenuation correction
MRE: Medically refractory epilepsy
MRI: Magnetic resonance imaging
PET: Positron emission tomography
PET/DTI: PET-guided diffusion tractography
RESOLUTE: Region specific optimization of continuous linear attenuation coefficients based on UTE
ROI: Region of interest
SEEG: Stereo-EEG
SPACE: Sampling perfection with application optimized contrasts using different flip angle evolution
SPM: Statistical parametric mapping
SUV: Standardized uptake value
SUVr: Relative SUV
VOI: Volume of interest
WM: White matter
Z_AI_: Z-score AI
