# Supplementary material for: 18F-FDG PET-guided diffusion tractography reveals white matter abnormalities around the epileptic focus in medically refractory epilepsy: implications for epilepsy surgical evaluation
Source: Eur J Hybrid Imaging. 2020 Jun 25;4:10. doi: 10.1186/s41824-020-00079-7 (PMC8218143; doi:10.1186/s41824-020-00079-7)
Supplement: Supplementary file 1 — Additional file 1: Figure S1. Diffusion MR image analysis pipeline. Table S1. EEG and MRI findings from clinical reports and visual assessment. Table S2. SUV analysis in hypometabolic PET ROIs and contralateral ROIs from AI mapping in 14 MRE patients. Table S3. Regional FA analysis in WM surrounding hypometabolic PET ROIs and contralateral ROIs detected by AI mapping of FDG-PET. [file 41824_2020_79_MOESM1_ESM.docx]

**SUPPORTING INFORMATION**

**
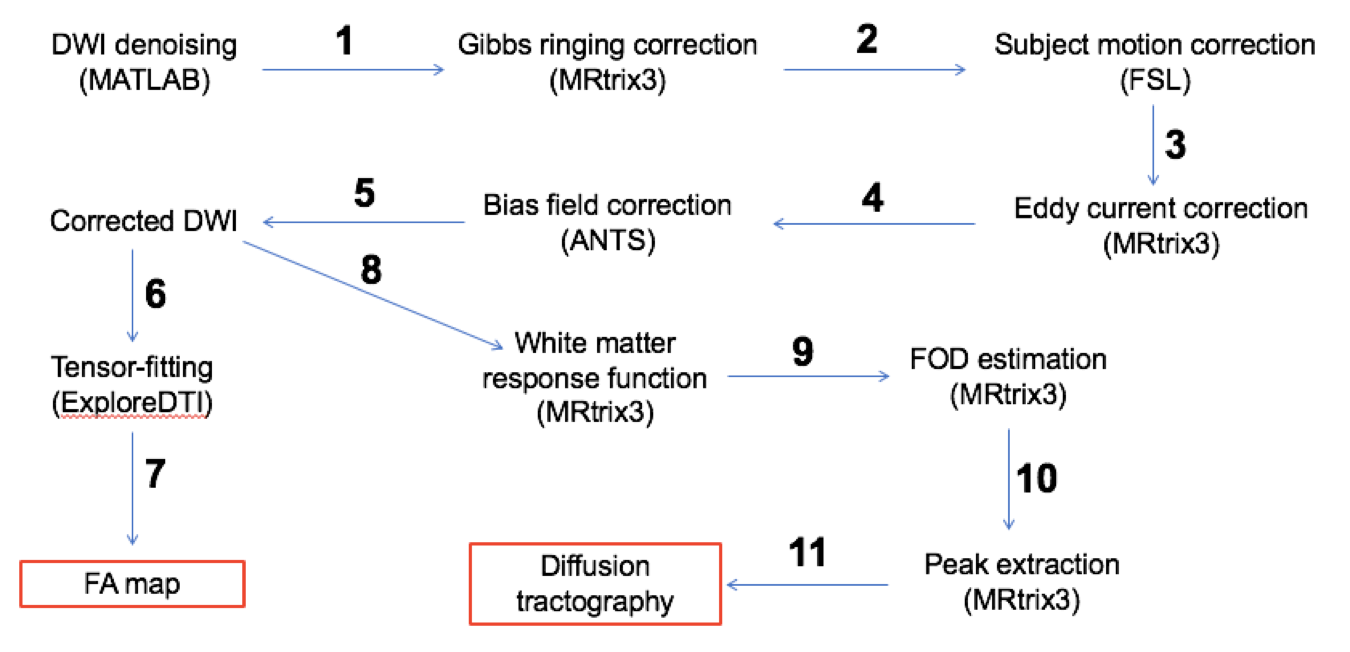
**

**Figure S1.** **Diffusion MR image analysis pipeline**. DWI data were preprocessed using a variety of different image processing software packages to generate an FA map and images that can be used to visualize WM fiber pathways in the brain using diffusion tractography. This pipeline was empirically evaluated using a ground-truth WM phantom from the ISMRM 2015 Tractography Challenge (Maier-Hein et al., 2017; Neher et al., 2014). Our pipeline scores were: viable bundles = 23/25, invalid bundles = 70, viable connections = 70.37%, invalid connections = 29.63%, and no connections = 0.00%. Comparing these scores to the online submissions database (http://www.tractometer.org/ismrm_2015_challenge/results), we found that our pipeline performed very well across all five scoring parameters and this gives us confidence that our pipeline is indeed accurately reconstructing WM fiber pathways in the brain.

Abbreviations: FOD = fiber orientation distribution.

**Table S1.** **EEG and MRI findings from clinical reports and visual assessment**

| **Patient No.** | **Clinical Hypothesis** | **Scalp EEG** | **1.5T MRI** | **3T MRI R1** | **3T MRI R2** | **AI Mapping** |
| --- | --- | --- | --- | --- | --- | --- |
| 1 | L temp/front | L temp/front | L MTS* | L MTS | L MTS | L temp |
| 2 | L front | Bifrontal | L temp FCD* | L front/par FCD | L front/par FCD | L front |
| 3 | R front | Bifrontal | Unremarkable | Unremarkable | Unremarkable | L front |
| 4 | L temp/front | L temp | Unremarkable | Unremarkable | Unremarkable | L temp |
| 5 | R temp | Bitemporal | Unremarkable | Unremarkable | Unremarkable | R temp |
| 6 | L temp/front | L temp/front | Unremarkable | Unremarkable | Unremarkable | L front |
| 7 | L temp | L temp/front | Unremarkable | Unremarkable | Unremarkable | L front |
| 8 | R front | Bitemporal + extratemporal | Bitemporal SH* | R MTS | Unremarkable | L front |
| 9 | L temp | L temp | L MTS* | Signal (L>R) | Unremarkable | L temp |
| 10 | R temp | R temp | Unremarkable | Unremarkable | Unremarkable | L front |
| 11 | R temp/front | R temp | Unremarkable | Unremarkable | Unremarkable | R temp |
| 12 | L temp | Bitemporal | Unremarkable | Unremarkable | Unremarkable | L temp |
| 13 | R temp | Bitemporal + extratemporal | Unremarkable | Unremarkable | Unremarkable | L temp/front |
| 14 | R temp | Bitemporal | Unremarkable | Unremarkable | Unremarkable | L temp |

Note. Findings from visual assessment of T1-weighted images from PET/MRI by two Neuroradiologists are summarized under the headings 3T MRI R1 and 3T MRI R2, while 1.5T MRI findings are from reports of clinically indicated MRI scans acquired prior to PET.

Abbreviations: *, equivocal finding; FCD, focal cortical dysplasia; front, frontal lobe; L, left; MTS, mesial temporal sclerosis; par, parietal lobe; R, right; SH, subcortical heterotopia; temp, temporal lobe.

**Table S2.** **SUV analysis in hypometabolic PET ROIs and contralateral ROIs from AI mapping in 14 MRE patients**

|  | **Mean SUV** | | **Max SUV** | | **Mean Z_AI_** | |
| --- | --- | --- | --- | --- | --- | --- |
| **Patient No.** | **Hypometabolic ROI** | **Contralateral ROI** | **Hypometabolic ROI** | **Contralateral ROI** | **Hypometabolic ROI** | **Contralateral ROI** |
| 1 | 2.24 | 3.16 | 3.11 | 4.10 | -2.30 | 2.02 |
| 2 | 4.90 | 8.00 | 5.89 | 9.45 | -2.10 | 1.93 |
| 3 | 4.81 | 6.82 | 6.41 | 7.86 | -2.70 | 2.40 |
| 4 | 1.63 | 2.16 | 2.06 | 2.69 | -2.20 | 1.83 |
| 5 | 3.03 | 3.94 | 4.17 | 5.46 | -2.07 | 1.69 |
| 6 | 5.35 | 7.48 | 6.68 | 8.75 | -2.34 | 1.96 |
| 7 | 2.01 | 2.70 | 4.05 | 4.78 | -3.21 | 2.85 |
| 8 | 4.66 | 8.14 | 8.29 | 10.85 | -3.42 | 3.09 |
| 9 | 2.83 | 4.04 | 3.89 | 5.08 | -2.66 | 2.31 |
| 10 | 3.87 | 5.43 | 5.10 | 6.60 | -2.73 | 2.37 |
| 11 | 5.87 | 8.09 | 7.67 | 11.16 | -2.12 | 1.87 |
| 12 | 4.95 | 6.73 | 7.25 | 9.69 | -2.53 | 2.19 |
| 13 | 6.16 | 7.98 | 8.58 | 10.68 | -2.33 | 1.95 |
| 14 | 4.34 | 5.78 | 5.00 | 6.46 | -2.71 | 2.35 |
| Group | 4.04 ± 1.47 | 5.75 ± 2.18 | 5.58 ± 2.00 | 7.40 ± 2.76 | -2.53 ± 0.41 | 2.20 ± 0.40 |

Note: For mean SUV and mean Z_AI_, group values are reported as mean ± standard deviation.

**Table S3.** **Regional FA analysis in WM surrounding hypometabolic PET ROIs and contralateral ROIs detected by AI mapping of FDG-PET**

|  | **3 mm into WM** | | **9 mm into WM** | | **15 mm into WM** | |
| --- | --- | --- | --- | --- | --- | --- |
| **Patient No.** | **Hypometabolic ROI** | **Contralateral ROI** | **Hypometabolic ROI** | **Contralateral ROI** | **Hypometabolic ROI** | **Contralateral ROI** |
| 1 | 0.08 | 0.18 | 0.15 | 0.12 | 0.16 | 0.13 |
| 2 | 0.07 | 0.17 | 0.11 | 0.19 | 0.12 | 0.16 |
| 3 | 0.14 | 0.13 | 0.26 | 0.22 | 0.28 | 0.24 |
| 4 | 0.08 | 0.14 | 0.12 | 0.14 | 0.13 | 0.16 |
| 5 | 0.06 | 0.05 | 0.08 | 0.06 | 0.08 | 0.07 |
| 6 | 0.10 | 0.15 | 0.15 | 0.26 | 0.19 | 0.27 |
| 7 | 0.01 | 0.02 | 0.03 | 0.04 | 0.06 | 0.05 |
| 8 | 0.08 | 0.17 | 0.11 | 0.16 | 0.13 | 0.20 |
| 9 | 0.12 | 0.19 | 0.22 | 0.26 | 0.24 | 0.25 |
| 10 | 0.12 | 0.18 | 0.21 | 0.21 | 0.22 | 0.20 |
| 11 | 0.07 | 0.14 | 0.16 | 0.22 | 0.15 | 0.19 |
| 12 | 0.05 | 0.10 | 0.12 | 0.09 | 0.10 | 0.09 |
| 13 | 0.13 | 0.13 | 0.19 | 0.18 | 0.18 | 0.18 |
| 14 | 0.13 | 0.16 | 0.24 | 0.29 | 0.26 | 0.31 |
| Group | 0.09 ± 0.04 | 0.13 ± 0.05 | 0.15 ± 0.06 | 0.17 ± 0.08 | 0.16 ± 0.07 | 0.18 ± 0.08 |

Note: Group values are reported as mean ± standard deviation.

**References**

Maier-Hein, K.H., Neher, P.F., Houde, J.-C., Côté, M.-A., Garyfallidis, E., Zhong, J., Chamberland, M., Yeh, F.-C., Lin, Y.-C., Ji, Q., Reddick, W.E., Glass, J.O., Chen, D.Q., Feng, Y., Gao, C., Wu, Y., Ma, J., Renjie, H., Li, Q., Westin, C.-F., Deslauriers-Gauthier, S., González, J.O.O., Paquette, M., St-Jean, S., Girard, G., Rheault, F., Sidhu, J., Tax, C.M.W., Guo, F., Mesri, H.Y., Dávid, S., Froeling, M., Heemskerk, A.M., Leemans, A., Boré, A., Pinsard, B., Bedetti, C., Desrosiers, M., Brambati, S., Doyon, J., Sarica, A., Vasta, R., Cerasa, A., Quattrone, A., Yeatman, J., Khan, A.R., Hodges, W., Alexander, S., Romascano, D., Barakovic, M., Auría, A., Esteban, O., Lemkaddem, A., Thiran, J.-P., Cetingul, H.E., Odry, B.L., Mailhe, B., Nadar, M.S., Pizzagalli, F., Prasad, G., Villalon-Reina, J.E., Galvis, J., Thompson, P.M., Requejo, F.D.S., Laguna, P.L., Lacerda, L.M., Barrett, R., Dell’Acqua, F., Catani, M., Petit, L., Caruyer, E., Daducci, A., Dyrby, T.B., Holland-Letz, T., Hilgetag, C.C., Stieltjes, B., Descoteaux, M., 2017. The challenge of mapping the human connectome based on diffusion tractography. Nature Communications 8, 1349. https://doi.org/10.1038/s41467-017-01285-x

Neher, P.F., Laun, F.B., Stieltjes, B., Maier-Hein, K.H., 2014. Fiberfox: Facilitating the creation of realistic white matter software phantoms: Realistic White Matter Software Phantoms. Magnetic Resonance in Medicine 72, 1460–1470. https://doi.org/10.1002/mrm.25045
